# Baroreflex sensitivity is blunted in hypoxia independently of changes in inspired carbon dioxide pressure in prematurely born male adults

**DOI:** 10.14814/phy2.15857

**Published:** 2024-01-03

**Authors:** Giorgio Manferdelli, Benjamin J. Narang, Nicolas Bourdillon, Tadej Debevec, Grégoire P. Millet

**Affiliations:** ^1^ Institute of Sport Sciences University of Lausanne Lausanne Switzerland; ^2^ Department of Automation, Biocybernetics and Robotics Jožef Stefan Institute Ljubljana Slovenia; ^3^ Faculty of Sport University of Ljubljana Ljubljana Slovenia

**Keywords:** altitude, baroreflex, hypercapnia, hypobaria, preterm

## Abstract

Premature birth may result in specific cardiovascular responses to hypoxia and hypercapnia, that might hamper high‐altitude acclimatization. This study investigated the consequences of premature birth on baroreflex sensitivity (BRS) under hypoxic, hypobaric and hypercapnic conditions. Seventeen preterm born males (gestational age, 29 ± 1 weeks), and 17 age‐matched term born adults (40 ± 0 weeks) underwent consecutive 6‐min stages breathing different oxygen and carbon dioxide concentrations at both sea‐level and high‐altitude (3375 m). Continuous blood pressure and ventilatory parameters were recorded in normobaric normoxia (NNx), normobaric normoxic hypercapnia (NNx + CO_2_), hypobaric hypoxia (HHx), hypobaric normoxia (HNx), hypobaric normoxia hypercapnia (HNx + CO_2_), and hypobaric hypoxia with end‐tidal CO_2_ clamped at NNx value (HHx + clamp). BRS was assessed using the sequence method. Across all conditions, BRS was lower in term born compared to preterm (13.0 ± 7.5 vs. 21.2 ± 8.8 ms⋅mmHg^−1^, main group effect: *p* < 0.01) participants. BRS was lower in HHx compared to NNx in term born (10.5 ± 4.9 vs. 16.0 ± 6.0 ms⋅mmHg^−1^, *p* = 0.05), but not in preterm (27.3 ± 15.7 vs. 17.6 ± 8.3 ms⋅mmHg^−1^, *p* = 0.43) participants, leading to a lower BRS in HHx in term born compared to preterm (*p* < 0.01). In conclusion, this study reports a blunted response of BRS during acute high‐altitude exposure without any influence of changes in inspired CO_2_ in healthy prematurely born adults.

## 1 INTRODUCTION

Premature birth (<37th week of gestation) is a growing health concern due to the increasing number of preterm survivors reaching adulthood reporting several prematurity‐related long‐term sequelae on the cardiovascular and respiratory systems (Duke et al., 2022; Lewandowski et al., 2013). Mounting evidence reports systemic and pulmonary artery hypertension in young adult survivors of premature birth (Alexander & Intapad, 2012; de Jong et al., 2012) leading to an increased risk of cardiovascular diseases and events during adulthood (Lewandowski et al., 2020; Markopoulou et al., 2019). Even though human investigations and animal models on the mechanisms by which premature birth per se leads to increased blood pressure (BP) remain limited, recent work demonstrated impaired vascular structure and function in young adults born preterm with high systolic blood pressure (SBP) in adult life (Lazdam et al., 2010). Moreover, increasing evidence demonstrates specific cardiovascular (Barton et al., 2021; Goss et al., 2015; Narang et al., 2022), respiratory (Bates et al., 2014; Debevec et al., 2019, 2022b; Duke et al., 2014; Farrell et al., 2015; Narang et al., 2022) and molecular (Martin et al., 2018 and 2020) responses to both hypoxia and hypercapnia in prematurely born children and young adults. While some authors reported beneficial effects of preterm birth during acute hypoxia exposure (Farrell et al., 2015; Goss et al., 2015), others suggested exaggerated and/or impaired responses to a reduced oxygen (O_2_) availability (Barton et al., 2021; Bates et al., 2014; Debevec et al., 2019) or increased CO_2_ breathing (Manferdelli et al., 2021; Rigatto et al., 1975).

The autonomic nervous system (ANS) has been extensively implicated in the onset of several cardiovascular diseases (Bigger JT Jr & PJ, 1994; Lown & Verrier, 1976) and growing evidences indicate reduced vagal activity, and thus relative sympathetic dominance (i.e., autonomic imbalance), as a potential pathway to increased morbidity and mortality from cardiovascular disease (Thayer et al., 2010). Limited results also suggest autonomic dysfunction in adult survivors of premature birth (Haraldsdottir et al., 2018; Karvonen et al., 2019; Mathewson et al., 2015) and therefore raise questions about its contribution in the higher risk of cardiac diseases in this population.

Baroreflex sensitivity (BRS) is typically used to investigate the integrated function of the autonomic nervous and cardiovascular systems in humans (La Rovere et al., 1998). The baroreflex is a powerful BP regulatory mechanism that detects changes in BP and evokes reflex circulatory adjustments aiming to buffer BP changes (La Rovere et al., 1995). The sensitivity of the arterial baroreflex control or cardiac activity plays a pivotal role in human health, as demonstrated by the inverse relation between BRS and the risk of mortality after myocardial infarction (La Rovere et al., 1998) or conversely by the improved BRS after endurance training (Parati et al., 2001).

High‐altitude exposure is known to challenge both autonomic balance (Hainsworth et al., 2007) and BP regulation in humans (Bourdillon et al., 2017, 2018), with recent evidence attributing a central role of the ANS in the acute response and adaptation to hypobaric hypoxia (Bourdillon et al., 2017, 2018; Hainsworth et al., 2007). Acute hypoxia is a potent activator of sympathetic activity, leading to increased release of catecholamines, increases in heart rate (HR), and regional vasoconstriction (Hainsworth et al., 2007). Meanwhile, baroreceptor afferents principally counteract the hypoxia‐induced rise in BP by increasing/decreasing the activity of the parasympathetic and sympathetic branches of the ANS. Previous works demonstrated that spontaneous BRS decreases during both acute and prolonged exposures to altitude (Bourdillon et al., 2017, 2018). This resetting of BRS is clear above 4500 m while it is less evident for lower altitudes (Querido et al., 2011).

The aim of the present study was twofold: (a) to compare the spontaneous cardiovagal BRS at sea‐level and after 1 day of exposure to high‐altitude in term born and prematurely born adults, and (b) to investigate the effects of different combinations of hypobaric, hypoxic, and hypercapnic gas mixtures on spontaneous cardiovagal BRS in term born controls and prematurely born but otherwise healthy adults. We hypothesized that (1) Hypoxic exposure would impair BRS to the same extent in prematurely born and term born participants; and (2) Hypercapnia would induce a greater influence on BRS in prematurely born.

## 2 METHODS

### 2.1 Participants

Seventeen term born and 17 healthy preterm male participants volunteered and gave written informed consent to participate in this study. The preterm born participants were recruited via the National preterm birth register managed by the University Clinical Centre in Ljubljana, Slovenia using medical record screening and telephone/email‐based individual interviews. The inclusion criteria for the preterm group were gestational age ≤32 weeks, birth weight ≤1500 g, O_2_ therapy at birth, and absence of diagnosed bronchopulmonary dysplasia. The inclusion criteria for the term born participants were gestational age ≥38 weeks and birth weight ≥2500 g. Birth‐related inclusion criteria for all participants were checked and confirmed during the initial birth/medical record screening procedure conducted during recruitment. Exclusion criteria for all participants included permanent altitude residence (≥1000 m), cardiopulmonary, hematological and/or kidney disorders, chronic medication use, smoking, and altitude/hypoxia exposure (≥2000 m) within the last month prior to the study. Participants were matched for age and for fitness status (i.e., V̇O_2max_). The experimental protocol was preregistered at ClinicalTrials.gov (NCT04739904), approved by both the National Medical Ethics Committee of Slovenia (0120‐101/2016‐2) and the Aosta Hospital Ethical Committee, and it was performed according to the Declaration of Helsinki.

### 2.2 Experimental design and ascent protocol

Participants were tested in two different occasions (outlined below), one at sea level (295 m; Ljubljana, Slovenia; barometric pressure ~ 737 ± 2 mmHg) and the other at high‐altitude (3375 m; Torino hut, Aosta Valley, Italy, on the Mont Blanc massif; barometric pressure ~ 503 ± 3 mmHg). Participants reached Torino hut from Courmayeur (1300 m) by cable car in about 15–20 min. At altitude, tests were performed in the first morning after arrival at altitude. Sea‐level testing was performed between 2 and 3 months before ascending to altitude. Detailed description of the general experimental design of the project was previously published elsewhere (Manferdelli et al., 2023a).

### 2.3 Experimental protocol

Before each trial, the participants were instructed to abstain from exercise for 12 h, avoid alcohol and caffeine for 24 h, and they did not consume a heavy meal within 4 h before testing. All experiments were conducted in the morning (9:00–11:00 a.m.) at the same time of the day with the participant comfortably seated on a chair. Following 10–15 min of quiet rest in seated position, each testing session included equipment instrumentation and six 6‐min stages of continuous BP, HR, and gas exchanges recording in the following order: (1) normobaric normoxia (NNx), (2) normobaric normoxia hypercapnia (NNx + CO_2_), (3) hypobaric hypoxia (HHx), (4) hypobaric normoxia (HNx), (5) hypobaric normoxia hypercapnia (HNx + CO_2_), (6) hypobaric hypoxia with P_ET_CO_2_ clamped at NNx value (HHx + clamp). Normobaric conditions were performed at sea level, while hypobaric measurements were carried out at high‐altitude. NNx + CO_2_ was induced by switching the inspired gas from ambient air to 3% CO_2_ (in 20.93% O_2_, balance N_2_). In HNx and HNx + CO_2_, participants breathed supplemental O_2_ (FiO_2_ = 32%, with 0.03% CO_2_, balance N_2_ and with 3% CO_2_, balance N_2_, respectively). This O_2_ concentration was calculated in order to induce the same inspired O_2_ partial pressure that participants breathed during NNx. During condition 6, end‐tidal clamping was performed using a modified version of the system developed by Olin et al. (2012). Briefly, the system is designed to deliver high‐flow, low‐resistance inspired gas with a fixed FiO_2_, and a varying inspired fraction of CO_2_ (FiCO_2_). The inspiratory endpoint of this system included an open‐ended reservoir where room air was mixed with 100% CO_2_ compressed gas. The 8‐L custom‐made reservoir was connected, via a plastic flexible tube, to a 2‐way non rebreathing valve (Hans Rudolph, 2700 series, Hans Rudolph, Kansas City, MO, USA) which was attached a low dead‐space face mask (Hans Rudolph mask, 7400 oronasal series; dead space, 73 mL).

### 2.4 Measurements

#### 2.4.1 Blood pressure and heart rate

Beat‐to‐beat systolic, diastolic, and mean arterial BP (SBP, DBP, and MAP, respectively), as well as HR, were monitored noninvasively using a finger photopletismography device (NIBP100D, Biopac Systems Inc., Goleta, CA, USA) combined to a double cuff installed on the index and the middle fingers. Automatic calibration of the device was performed immediately before the start of each test by measuring BP on the left arm of the participant at the level of the brachial artery using a cuff.

#### 2.4.2 Respiratory gas exchange parameters

Participants breathed trough a leak‐free respiratory mask (Hans‐Rudolph 7450 series) attached to a T‐shaped two‐way non‐rebreathing valve (see *Experimental protocol*). P_ET_CO_2_, pulmonary ventilation (V̇_E_), tidal volume (V_T_), and breathing frequency (B_*f*_) were measured breath‐by‐breath and recorded using a metabolic cart (Ergocard Professional, Medisoft, Sorinnes, Belgium).

#### 2.4.3 Arterialized capillary blood gas variables

For each participant, capillary blood samples were taken from the earlobe during the last 30 s of each stage. Arterialization of capillary blood (McEvoy & Jones, 1975), aiming to induce a capillary shunting between arterial and venous territories, was achieved by applying a vasodilation cream (Capsolin, SIT s.r.l., Mede, Italy). Arterial blood gas parameters, including partial pressure of O_2_ (P_a_O_2_), partial pressure of CO_2_ (P_a_CO_2_), pH, hydrogen ion concentration ([H^+^]), bicarbonate concentration ([HCO_3_^−^]), base excess, and arterial oxygen saturation (S_a_O_2_), were immediately analyzed using an arterial blood gas analyzer (ABL‐90 FLEX, Radiometer, Copenhagen, Denmark). All samples were heated/corrected to an assumed resting body temperature of 37.0°C.

#### 2.4.4 Heart rate variability

Heart rate variability (HRV) was assessed in the supine position upon awake both at sea‐level and on the first morning following arrival at high‐altitude, with empty bladder, and fasting. Participants were instructed to breathe spontaneously. The inter‐beat interval (R‐R interval) was measured for 5 min using a chest strap (Polar H7, Polar, Kempele, Finland) which was connect via Bluetooth to participants' smartphones (mobile application: inCORPUS v2.4.1, be. care SA, Renens, Switzerland). HRV analyses were performed using the Welch power spectral density estimate in the low‐frequency (LF, 0.04–0.15 Hz; in ms^2^) and high‐frequency bands (HF, 0.15–0.40 Hz; in ms^2^) and the root mean square of the successive differences (RMSSD; in ms) (Heart rate variability: Standards of measurement, physiological interpretation and clinical use, 1996). Briefly, LF holds information on both the sympathetic and parasympathetic branches and mainly reflects the baroreflex activity, whereas HF reflects parasympathetic activity and is mainly associated with respiratory sinus arrythmia. RMSSD provides information on vagally mediated changes in HR (Shaffer & Ginsberg, 2017).

#### 2.4.5 Acute mountain sickness

Symptoms of acute mountain sickness (AMS) were assessed in the morning of the test upon awake (about 16 h after arrival at altitude) using the 2018 Lake Louise Scale (Roach et al., 2018). Accordingly, AMS was defined when headache was present together with at least one additional symptom, and the total score was three or higher.

### 2.5 Data acquisition

Cardiovascular data were measured continuously at 1000 Hz using an analog‐to‐digital converter (MP150, Biopac Systems Inc, Goleta, CA, USA, respectively). The device was interfaced to a PC using a dedicated software (Acknowledge v.4.2, Biopac Systems Inc, Goleta, CA, USA, respectively), and data were stored on computer for later off‐line analyses. Signal processing was performed using custom Matlab routines (MATLAB, R2020b, MathWorks, Natick, MA, USA).

#### 2.5.1 Data exclusion

Data from one subject were excluded from statistical analyses due to three fainting episodes (one in NNx and the others in HHx). Moreover, BP data from one participant at altitude were not available because of loss of signal from the photopletismography device. Therefore, overall, 16 term born and 17 preterm successfully completed stages 1–2, while 15 term born and 17 preterm completed stages 3–5. In addition, only 10 term born and 6 preterm were able to complete the 6 min of the HHx + clamp phase.

### 2.6 Data analyses

Respiratory variables for each condition were calculated as the average of the last 30 s of each stage. The hypercapnic ventilatory response (HCVR) at sea level (Stages 1 and 2 of the experimental protocol) was calculated as the ratio between the delta in V̇_E_ and the delta in P_ET_CO_2_. SBP peaks were initially extracted from the BP waveform with heart beats representing the time of their occurrence and heart beat‐to‐beat time intervals (inter‐beat interval, IBI) was extracted directly from BP recordings and calculated as the interval between successive systolic peaks. A second order polynomial was interpolated for each extracted peak using four neighbor samples (two immediately before and two immediately after) from the BP waveform in order to refine the heartbeats placement. Heartbeats were selected as the location of the maximum of the interpolated polynomial (Bourdillon et al., 2018). Also, SBP values were updated as the maximum in their corresponding polynomial, and the IBI were created as the interval between successive peaks. BRS was then calculated using the sequence method, as previously described (Bourdillon et al., 2018). Briefly, this method of analysis provides a direct interpretation of the causal link between BP and HR (Parati et al., 1988), and it was shown to be highly reliable (Pinna et al., 2015). This method identifies at least three consecutive beats in which a strictly defined increase (or decrease) in SBP is followed by a strictly defined increase (or decrease) in the IBI. Fixed maximal changes were considered for SBP and IBI in order to consider the sequence valid. Specifically, a minimum change of 1 mmHg between two consecutive SBP peaks and or at least 5 ms change in a sequence (Bernardi et al., 2010). In addition, to accept the sequence, the minimum correlation coefficient between changes in SBP and IBI was 0.85. Finally, a minimum number of five sequences was set to validate a BRS estimate. The sensitivity of the baroreflex was obtained by computing the slope of the regression line between changes in SBP and IBI, and subsequently all the computed slopes were averaged to obtain the BRS. BRS was thus calculated using a 6‐min window for each stage.

### 2.7 Statistical analysis

The number of recruited participants was established on a priori calculation (*α* = 0.05, *β* = 0.80) based on previous works from our research group (Bourdillon et al., 2017, 2018) investigating BRS in the general population under the influence of different environmental stimuli (i.e., hypobaria, hypoxia, hypercapnia).

**FIGURE 1:**
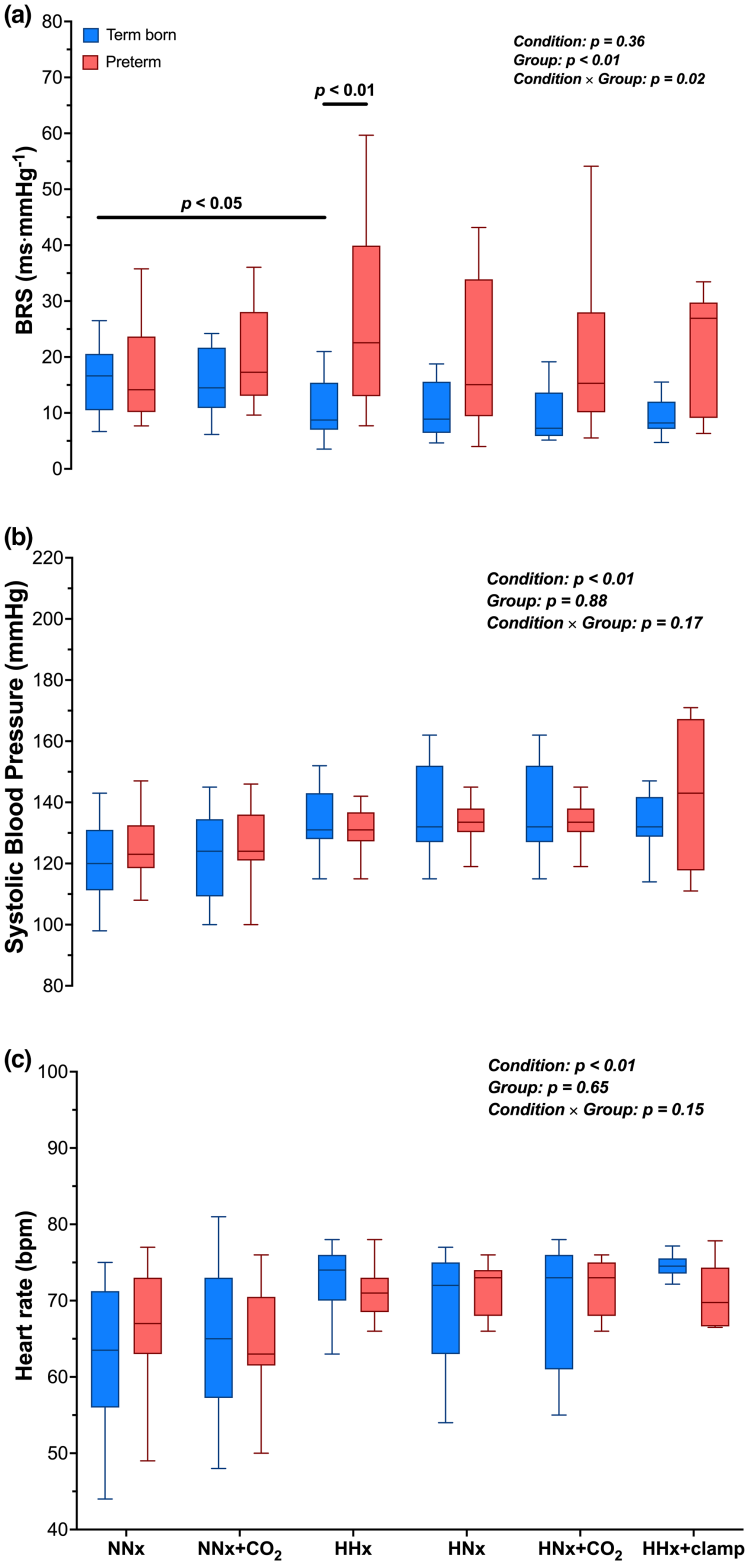
Tukey boxplots, horizontal line inside boxes: median; upper and lower lines of boxes: 75th and 25th percentiles, respectively; upper and lower whiskers: highest and lowest data points within the 1.5 inter quartile range. Baroreflex sensitivity (BRS; Panel a), systolic blood pressure (SBP; Panel b), and heart rate (HR; Panel c) in term born (in blue) and prematurely born (in red) adults during the 6 stages of the experimental protocol. NNx, normobaric normoxia (*n* = 33); NNx + CO_2_, normobaric normoxia with 3% CO_2_ (*n* = 33); HHx, hypobaric hypoxia (3375 m; *n* = 32); HNx, hypobaric normoxia (*n* = 32); HNx + CO_2_, hypobaric normoxia with 3% CO_2_ (*n* = 32); HHx + clamp, hypobaric hypoxia with P_ET_CO_2_ clamped at NNx value (*n* = 16).

All data are presented as mean ± SD throughout the manuscript. Figure 1 display BRS, SBP, and HR, in Tukey boxplots in which the horizontal line inside the boxes is the median, while the upper and lower lines of the boxes represent the 75th and 25th percentiles, respectively. The upper and lower whiskers denote the highest and lowest data points within the 1.5 inter quartile range which corresponds to approximately 2.7 s and 99.3% coverage of the data.

A two‐way (Group × Condition) repeated measures ANOVA was performed to compare BRS, respiratory gas exchange parameters, HRV indices, and arterial blood gas variables between term born and preterm participants. Significant main and interaction effects were analyzed by Sidak correction. After having checked for normality, HCVR was analyzed by unpaired Student *t*‐test. AMS prevalence was analyzed by Fisher's exact test. Effect sizes were calculated using Cohen's *d* and reported along 95% confidence intervals (95% CI) (Nakagawa & Cuthill, 2007). The effect size was considered negligible when *d* < 0.2, small when *d* ≥ 0.2, moderate when *d* ≥ 0.5, large when *d* ≥ 0.8, and very large when *d* ≥ 1.3 (Sullivan & Feinn, 2012). All *p*‐values are two‐tailed and statistical significance was defined a priori at *p* < 0.05. Data analyses was performed using the statistical software package Prism v.8.0 (GraphPad Software, San Diego, CA, USA).

## 3 RESULTS

Table 1 shows participants' physical characteristics. Mild AMS was present in two term born and four preterm participants at high‐altitude. However, no difference in AMS prevalence was found between groups (*p* = 0.656). In both term born and preterm participants, inspired O_2_ pressure was matched between NNx versus HNx versus HNx + CO_2_ (142.9 ± 0.5 vs. 139.0 ± 3.7 vs. 139.8 ± 2.5 mmHg, respectively). Likewise, P_ET_CO_2_ was matched between NNx and HHx + clamp (38 ± 3 vs. 36 ± 3 mmHg, respectively).

**TABLE 1:** Participants' physical characteristics (Mean ± SD).

	Term born	Preterm	*p*‐value
Participants' characteristics			
Gestational age (weeks)	40 ± 1	29 ± 2	***p* < 0.01**
Birth weight (g)	3621 ± 421	1132 ± 265	***p* < 0.01**
Age (years)	21 ± 2	21 ± 4	*p* = 0.07
Height (cm)	182 ± 6	178 ± 9	*p* = 0.21
Body mass (kg)	75.6 ± 6.9	72.4 ± 14.4	*p* = 0.42
BMI (kg·m^−2^)	22.8 ± 1.8	22.5 ± 2.7	*p* = 0.74
V̇O_2peak_ (mL·kg^−1^·min^−1^)	51.3 ± 7.6	48.5 ± 10.7	*p* = 0.40

*Note*: *p* value in bold indicates statistical significance.

Abbreviation: BMI, body mass index.

**FIGURE 2:**
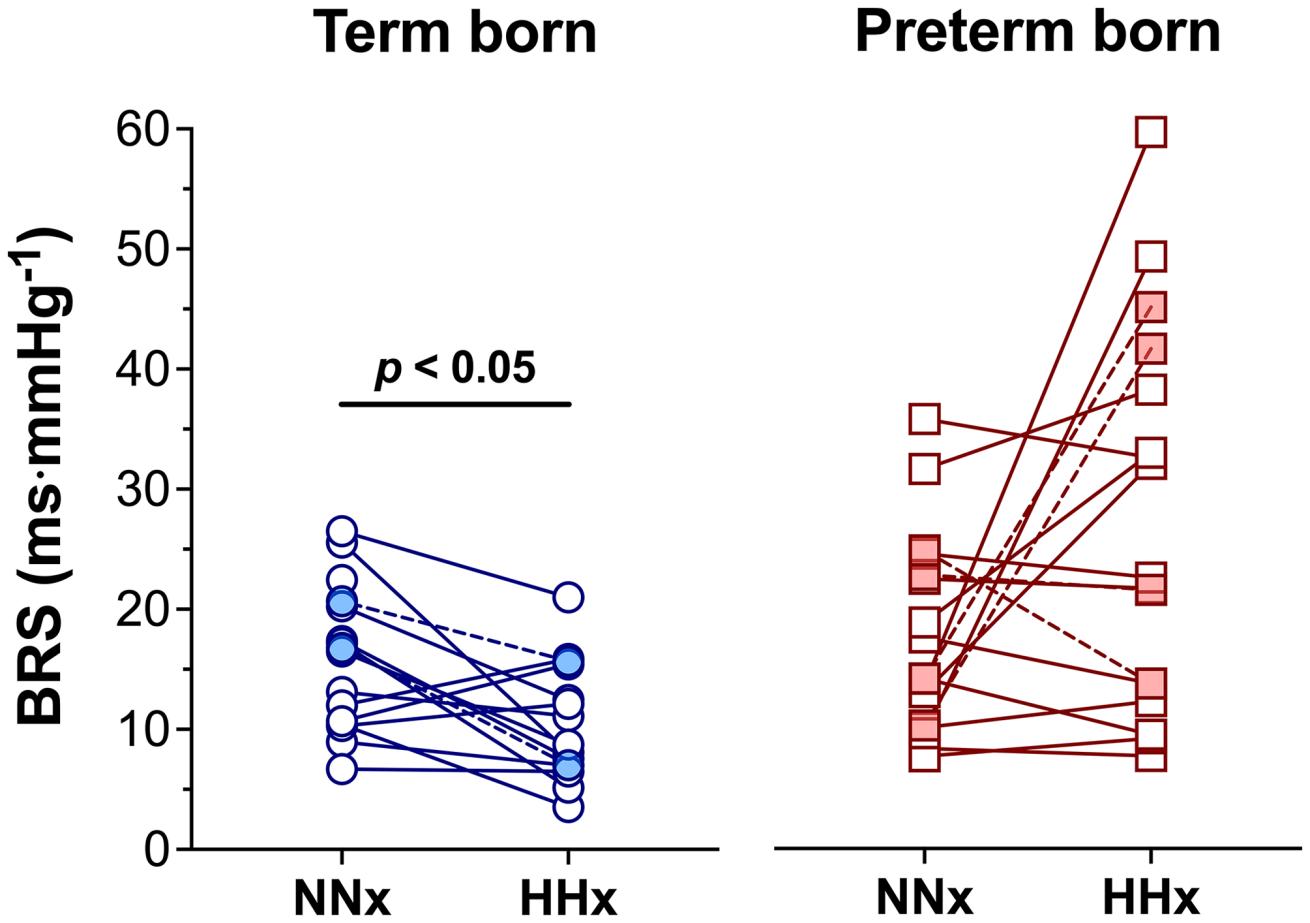
Individual changes in spontaneous cardiovagal baroreflex sensitivity (BRS) from normobaric normoxia (NNx) to hypobaric hypoxia (HHx) in term born (left panel) and preterm (right panel) adults. Filled symbols and dashed lines represent the changes in BRS from NNx to HHx in individuals with acute mountain sickness.

Figure 1 depicts BRS (panel a), SBP (panel b), and HR (panel c) in both groups during sstages 1–6 of the experimental protocol. Across all conditions, BRS was lower in term born compared to preterm (13.0 ± 7.5 vs. 21.2 ± 8.8 ms⋅mmHg^−1^, main group effect: *p* < 0.01) participants. Despite similar cardiorespiratory fitness (in terms of relative V̇O_2peak_—shown in Manferdelli et al. (2023a)), a lower BRS in HHx compared to NNx was observed in term born (10.5 ± 4.9 vs. 16.0 ± 6.0 ms⋅mmHg^−1^, respectively, *p* = 0.05, *d* = 1.0, 95% CI = −1.5 to −9.5), but not in preterm (27.3 ± 15.7 vs. 17.6 ± 8.3 ms⋅mmHg^−1^, *p* = 0.43) participants, leading to a lower BRS in HHx in term born compared to preterm (10.5 ± 4.9 vs. 27.3 ± 15.7 ms⋅mmHg^−1^, respectively, *p* < 0.01, *d* = 1.5, 95% CI = −5.0 to −28.5) adults. Individual changes in BRS from NNx to HHx in term born and preterm adults are shown in Figure 2. Of note, a significantly lower BRS at HHx in term born compared to preterm adults (*p* = 0.03) persisted even when participants reporting symptoms of AMS (two term born and four preterm adults) were removed from statistical analyses. V̇_E_, P_ET_CO_2_, B_*f*_, V_T_, MAP, and DBP recorded during Stages 1–6 of the experimental protocol are reported in Table 2. The ventilatory responses to hypoxia, hypobaria, and hypercapnia were similarly affected by each condition (*p* < 0.001) in the two groups (*p* = 0.303). Despite this and although we did not reach a statistical significance, HCVR in NNx was higher in preterm compared to term born (1.14 ± 0.72 vs. 0.75 ± 0.37 L⋅min^−1^⋅mmHg^−1^, *p* = 0.08) participants.

**TABLE 2:** Respiratory parameters, mean arterial pressure and diastolic blood pressure recorded during the six phases of the experimental protocol in term born and prematurely born participants.

Phase	Term born	Preterm	Group	Condition	Group × condition
V̇_E_ (L⋅min^−1^)					
NNx	11.5 ± 1.6	11.3 ± 2.0	*p* = 0.30	***p* < 0.01**	*p* = 0.10
NNx + CO_2_	15.0 ± 2.5	15.6 ± 3.2			
HHx	12.9 ± 1.7	13.1 ± 1.7			
HNx	12.6 ± 1.5	12.4 ± 1.9			
HNx + CO_2_	16.3 ± 1.7	16.2 ± 2.8			
HHx + clamp	20.2 ± 6.4	21.3 ± 5.2			
P_ET_CO_2_ (mmHg)					
NNx	38 ± 2	38 ± 3	*p* = 0.29	***p* < 0.01**	*p* = 0.95
NNx + CO_2_	43 ± 2	42 ± 3			
HHx	32 ± 2	31 ± 1			
HNx	33 ± 2	32 ± 2			
HNx + CO_2_	35 ± 1	35 ± 2			
HHx + clamp	36 ± 3	36 ± 4			
B_*f*_ (BPM)					
NNx	16 ± 4	15 ± 4	*p* = 0.97	***p* < 0.01**	*p* = 0.93
NNx + CO_2_	16 ± 3	15 ± 3			
HHx	17 ± 3	17 ± 4			
HNx	17 ± 3	17 ± 5			
HNx + CO_2_	18 ± 3	17 ± 4			
HHx + clamp	18 ± 4	19 ± 3			
V_T_ (L)					
NNx	0.7 ± 0.2	0.8 ± 0.2	*p* = 0.54	***p* < 0.01**	*p* = 0.97
NNx + CO_2_	1.0 ± 0.2	1.0 ± 0.3			
HHx	0.8 ± 0.1	0.8 ± 0.2			
HNx	0.7 ± 0.1	0.7 ± 0.2			
HNx + CO_2_	0.9 ± 0.1	1.0 ± 0.1			
HHx + clamp	1.1 ± 0.2	1.1 ± 0.2			
MAP (mmHg)					
NNx	84 ± 9	86 ± 8	*p* = 0.99	***p* < 0.01**	*p* = 0.63
NNx + CO_2_	85 ± 10	86 ± 10			
HHx	94 ± 8	92 ± 8			
HNx	94 ± 9	93 ± 9			
HNx + CO_2_	94 ± 10	94 ± 9			
HHx + clamp	95 ± 8	97 ± 14			
DBP (mmHg)					
NNx	66 ± 9	66 ± 9	*p* = 0.93	***p* < 0.01**	*p* = 0.90
NNx + CO_2_	66 ± 9	66 ± 10			
HHx	74 ± 9	72 ± 9			
HNx	73 ± 9	73 ± 9			
HNx + CO_2_	72 ± 9	74 ± 9			
HHx + clamp	75 ± 7	75 ± 13			

*Note*: *p* value in bold indicates statistical significance.

Abbreviations: B_*f*_, breathing frequency; DBP, diastolic blood pressure; HHx + clamp, hypobaric hypoxia with end tidal partial pressure of CO_2_ (P_ET_CO_2_) clamped at NNx value; HHx, hypobaric hypoxia; HNx + CO_2_, hypobaric normoxia with 3% CO_2_; HNx, hypobaric normoxia; MAP, mean arterial pressure; NNx + CO_2_, normobaric normoxia with 3% carbon dioxide (CO_2_); NNx, normobaric normoxia; V̇_E_, pulmonary ventilation, V_T_, tidal volume.

Arterial blood gas parameters collected during each stage are listed in Table 3.

**TABLE 3:** Arterial blood gas variables in term born and prematurely born participants collected during the last 30 s of each phase of the experimental protocol.

Phase	Term born	Preterm	Group	Condition	Group × condition
PaO_2_ (mmHg)					
NNx	87 ± 9	87 ± 10	*p* = 0.63	***p* < 0.01**	*p* = 0.80
NNx + CO_2_	98 ± 6	101 ± 6			
HHx	53 ± 2	53 ± 3			
HNx	87 ± 5	88 ± 6			
HNx + CO_2_	99 ± 3	100 ± 4			
HHx + clamp	66.3 ± 3.9	68.1 ± 1.8			
PaCO_2_ (mmHg)					
NNx	42.4 ± 2.1	40.9 ± 3.3	*p* = 0.26	***p* < 0.01**	*p* = 0.23
NNx + CO_2_	44.2 ± 1.7	43.6 ± 3.3			
HHx	34.4 ± 1.3	33.6 ± 2.9			
HNx	36.5 ± 1.2	35.5 ± 2.5			
HNx + CO_2_	37.6 ± 1.1	36.9 ± 2.3			
HHx + clamp	37.4 ± 2.7	39.2 ± 3.3			
pH					
NNx	7.41 ± 0.01	7.41 ± 0.02	*p* = 0.20	***p* < 0.01**	*p* = 0.32
NNx + CO_2_	7.40 ± 0.02	7.39 ± 0.02			
HHx	7.44 ± 0.02	7.44 ± 0.01			
HNx	7.43 ± 0.02	7.42 ± 0.02			
HNx + CO_2_	7.42 ± 0.02	7.41 ± 0.02			
HHx + clamp	7.42 ± 0.02	7.40 ± 0.00			
[H^+^] (nM)					
NNx	38.6 ± 1.3	38.8 ± 2.0	*p* = 0.13	***p* < 0.01**	*p* = 0.15
NNx + CO_2_	39.7 ± 1.8	40.8 ± 2.3			
HHx	36.0 ± 1.4	36.0 ± 1.2			
HNx	37.4 ± 1.6	37.8 ± 1.6			
HNx + CO_2_	38.3 ± 1.6	38.6 ± 1.4			
HHx + clamp	37.9 ± 2.2	40.1 ± 0.5			
[HCO_3_^−^] (mEq⋅L^−1^)					
NNx	26.7 ± 1.8	25.7 ± 2.5	*p* = 0.10	***p* < 0.01**	*p* = 0.90
NNx + CO_2_	27.2 ± 1.5	26.1 ± 2.4			
HHx	23.6 ± 1.1	23.0 ± 1.7			
HNx	24.1 ± 1.2	23.1 ± 1.5			
HNx + CO_2_	24.2 ± 1.1	23.6 ± 1.5			
HHx + clamp	24.4 ± 1.1	24.1 ± 1.7			
SaO_2_ (%)					
NNx	96.6 ± 1.1	96.5 ± 1.8	*p* = 0.62	***p* < 0.01**	*p* = 0.97
NNx + CO_2_	98.0 ± 0.4	98.2 ± 0.4			
HHx	88.6 ± 1.7	89.0 ± 1.8			
HNx	97.2 ± 0.9	97.4 ± 0.5			
HNx + CO_2_	98.1 ± 0.4	98.2 ± 0.4			
HHx + clamp	93.3 ± 1.1	93.9 ± 1.3			

*Note*: *p* value in bold indicates statistical significance.

Abbreviations: [H^+^], hydrogen concentration; [HCO_3_^−^], bicarbonate concentration; HHx + clamp, hypobaric hypoxia with end tidal partial pressure of CO_2_ (P_ET_CO_2_) clamped at NNx value; HHx, hypobaric hypoxia; HNx + CO_2_, hypobaric normoxia with 3% CO_2_; HNx, hypobaric normoxia; NNx + CO_2_, normobaric normoxia with 3% carbon dioxide (CO_2_); NNx, normobaric normoxia; PaCO_2_, arterial partial pressure of CO_2_; PaO_2_, arterial partial pressure of oxygen (O_2_); SaO_2_, arterial O_2_ saturation.

Both HF and LF were similar between groups (term born: 2175.3 ± 988.8 ms^2^ and 1416.5 ± 1137.8 ms^2^; preterm: 2081.4 ± 1009.3 ms^2^ and 1777.3 ± 1172.6 ms^2^; main effect of group: *p* = 0.942 and *p* = 0.345, respectively) and conditions (sea level: 2445.5 ± 1475.1 ms^2^ and 1642.3 ± 1284.9 ms^2^; high‐altitude: 1692.8 ± 1216.1 ms^2^ and 1566.2 ± 1416.5 ms^2^; main effect of condition: *p* = 0.451 and *p* = 0.861, respectively). RMSSD was similar between term born and preterm participants (73.6 ± 18.6 vs. 68.6 ± 16.1 ms; main effect of group: *p* = 0.683), and it was decreased by hypoxia in both groups (80.1 ± 19.6 to 57.7 ± 23.6 ms; main effect of condition: *p* < 0.001).

## 4 DISCUSSION

We investigated BRS at sea level and high‐altitude in prematurely born adults and age‐matched term born controls to tease out any differential responses in the two cohorts. The main findings were (1) BRS was reduced by acute exposure to hypobaric hypoxia in term born adults, but not in their preterm peers, leading to a significantly lower BRS at altitude in this later group; and (2) Hypo‐ or hyper‐capnic conditions, did not provoke any specific differences between the two groups. Collectively, these results provide evidence of a blunted BRS during hypoxic exposure, independently of changes in inspired CO_2_, in preterm born adults.

The hypoxia‐induced decrease in BRS observed among the term born adults investigated in the present study confirmed well‐established observations (Bourdillon et al., 2017, 2018); and the mechanisms by which BRS is decreased have been already described. Briefly, hypoxia attenuates the parasympathetic drive to the heart which leads to a resetting of BRS to higher BP values (Querido et al., 2011). This response to acute hypoxia occurs in parallel with hypoxia‐induced overactivity of the peripheral chemoreceptors which results in a coordinated response between the baro‐ and chemo‐reflexes (Purves et al., 2001). Overall, this integrated mechanism underlies the well‐known sympathoexcitation occurring during acute hypoxic exposure (Querido et al., 2011).

Intriguingly, preterm adults demonstrated a blunted baroreflex response to hypoxia, which suggests a dysfunction in this integrated mechanism. The higher, compared to term born controls, and unchanged, compared to normoxia, BRS found in prematurely born adults in hypoxia is a new finding that was not yet described in the literature. Although data on autonomic function in premature birth survivors beyond infancy is limited, emerging evidence suggests impaired autonomic function in adolescents (Haraldsdottir et al., 2018) and young adults born preterm (Karvonen et al., 2019; Mathewson et al., 2015). Some authors suggested reduced parasympathetic activity in adults born prematurely (Karvonen et al., 2019; Mathewson et al., 2015). However, in the present study, HRV indices of sympathetic and parasympathetic activity at rest were similar between preterm and term born adults, thus suggesting—with caution due to the indirect nature of HRV—a normal autonomic function in the former population. Another potential explanation might relate to the adverse effects induced by postnatal hyperoxic treatment commonly applied to premature infants. As briefly outlined above, cardiac autonomic control is largely dictated by both baro‐ and chemo‐receptors feedbacks (Purves et al., 2001), and animal models demonstrated lower carotid body density and reduced sensitivity of the afferent limb of the arterial chemoreceptor reflex in rats exposed to postnatal hyperoxia (Bisgard et al., 2003). These anatomical and functional deficits were suggested to impair the normal baroreflex growth and sensitivity in infants born preterm (Witcombe et al., 2012), and thus to impair autonomic responses later into adulthood (Haraldsdottir et al., 2018). While we did not observe lower BRS in our healthy preterm cohort compared to term born peers under normoxic conditions, the blunted hypoxic chemosensitivity previously reported in the former cohort (Bates et al., 2014; Debevec et al., 2019) might cause or go in parallel with a blunted baroreceptors response to a hypoxic stimulus. However, a parallel data collection during a previous study demonstrated similar hypoxic ventilatory response in preterm adults and age‐matched controls born at term (Manferdelli et al., 2023a). The difference between our previous published data and previous studies in the literature on hypoxic chemosensitivity in preterm population is likely due to methodological differences in assessing the hypoxic ventilatory response.

Reduced BRS is a well‐known risk factor for cardiovascular mortality and morbidity. Even though the present study is the first to demonstrate an unchanged BRS during acute hypoxic exposure in preterm adults, our results may reinforce previous speculations (Debevec, Narang, et al., 2022) of increased susceptibility to high‐altitude illnesses in this population. Emerging evidence supports specific phenotypical responses to hypoxia at cardiovascular level in prematurely born adults. Barton and colleagues recently demonstrated an exaggerated cardiac contractile response, especially at the level of the right ventricle, in healthy preterm adults acutely exposed to severe hypoxia (FiO_2_ = 12%) and concluded that exposure to hypoxia may ultimately increase their risk for late right ventricle heart failure (Barton et al., 2021). We also recently confirmed impaired microvascular responsiveness in preterm adults, compared to term born controls, upon arrival at high‐altitude (3375 m) (Manferdelli et al., 2023b), thus further suggesting specific cardiovascular responses to a physiological stressor, such as hypoxia, in this population.

Additional aim of the present work was also to elucidate the effects of breathing different O_2_ and CO_2_ mixtures on BRS in both preterm and term born adults to understand the respective contribution of hypoxic versus hypo‐ or hyper‐capnia stimuli that are known to differently affect chemoreflex activation. Recent well‐designed studies demonstrated that BRS is reduced during acute exposure to hypobaric hypoxia, though this decrease was counterbalanced by breathing either supplemental O_2_ (Yazdani et al., 2016) or additional CO_2_ (Bourdillon et al., 2018). Our findings confirm these previous results and provide further evidence that the BRS decrease occurring in acute hypoxia is partially mediated by carotid body chemoreceptors (Mozer et al., 2016). Earlier studies observed a persistent increase in chemoafferent activity to the rostroventrolateral medulla via the nucleus tractus solitarius during acute hypoxia, which results in long‐lasting sympathoexcitation (Guyenet, 2000; Prabhakar & Kumar, 2010). Consequently, hypoxia‐induced hypocapnia was shown to deactivate chemoreceptors activity leading to decreased BRS (Querido et al., 2011), while either breathing 3% hypercapnic mixture or normalizing P_ET_CO_2_ to sea level values (during HHx + clamp) restored BRS as in NNx likely because the sensitivity of the peripheral chemoreceptors remained unchanged. Likewise, breathing mild hyperoxic gas mixture significantly reduced their contribution and restored BRS to NNx values.

### 4.1 Methodological considerations

Although the present study provides novel insight into the autonomic cardiac regulation in this preterm population, there are some considerations to be acknowledged. First the tested cohorts have been comprised of male participants solely. Accordingly, these results cannot be directly extended to female individuals. Despite emerging evidences from rodent studies suggesting sex‐specific differences in the preterm population (Tetri et al., 2018) further work on this topic is urgently needed. Second, respiratory sinus arrythmia is a known confounding factor for the characterization of spontaneous baroreflex control at rest (Porta et al., 2012). Particularly, SBP shows a large oscillation at the respiratory frequency (Karemaker, 2009a, 2009b), thus affecting the analyses of spontaneous cardiovagal BRS. However, as shown in Table 2 of the present study, the fact that no differences in ventilation or breathing frequency were noted between the two groups suggests that respiratory sinus arrythmia unlikely confounded the present outcomes. Also, BRS was assessed only by using the sequence method. However, this is the most common method, which allows a direct interpretation of the causal link between BP and HR changes (Parati et al., 1988). Finally, the activity of the ANS was evaluated indirectly using HRV indices recorded by HR monitor, rather than the gold‐standard ECG. However, the use of a chest strap was previously shown to provide comparable results to ECG‐based HRV monitoring (Plews et al., 2017).

## 5 CONCLUSION

This study reported a different spontaneous cardiovagal BRS response to acute hypoxic exposure in prematurely born adults, compared to term born controls. Prematurely but otherwise healthy adults showed a blunted hypoxia‐induced resetting of spontaneous cardiovagal BRS, independently of the changes in inspired carbon dioxide pressure. The clinical application of this blunted baroreflex resetting in acute hypoxia requires further application in adults born prematurely exposed to prolonged high‐altitude (e.g., mountaineers).

## AUTHOR CONTRIBUTIONS

Grégoire P. Millet and Tadej Debevec were involved in conceived the research and obtained the financial support. Giorgio Manferdelli, Nicolas Bourdillon, and Grégoire P. Millet were involved in contributed to the experimental design. Giorgio Manferdelli and Benjamin J. Narang were involved in collected the data. Giorgio Manferdelli, Nicolas Bourdillon, and Grégoire P. Millet were involved in analyzed and interpreted the data. Giorgio Manferdelli was involved in drafted the manuscript. All authors critically revised the draft and approved the final version of the manuscript.

## FUNDING INFORMATION

This work was funded by the Swiss National Science Foundation (SNSF grant nr. 320030L_192073) and the Slovenian Research Agency (ARRS grant nr. N5‐0152).

## CONFLICT OF INTEREST STATEMENT

No conflicts of interest, financial or otherwise, are declared by the authors.

## ETHICS STATEMENT

The experimental protocol was pre‐registered at ClinicalTrials.gov (NCT04739904), approved by both the National Medical Ethics Committee of Slovenia (0120‐101/2016‐2) and the Aosta Hospital Ethical Committee, and it was performed according to the Declaration of Helsinki.

## Data Availability

The data that support the findings of this study are available from the corresponding author upon reasonable request.
